# Gas-Phase Chemistry of 1,1,2,3,3,4,4-Heptafluorobut-1-ene Initiated by Chlorine Atoms

**DOI:** 10.3390/molecules27030647

**Published:** 2022-01-19

**Authors:** Ramesh Sapkota, Paul Marshall

**Affiliations:** 1 Department of Chemistry, University of North Texas, 1155 Union Circle, #305070, Denton, TX 76203, USA; RameshSapkota@my.unt.edu; 2 Center for Advanced Scientific Computing and Modeling, University of North Texas, 1155 Union Circle, #305070, Denton, TX 76203, USA

**Keywords:** hydrofluorocarbon, GWP, infrared spectroscopy, kinetics, atmospheric chemistry

## Abstract

The possibility of mitigating climate change by switching to materials with low global warming potentials motivates a study of the spectroscopic and kinetic properties of a fluorinated olefin. The relative rate method was used to determine the rate constant for the reaction of heptafluorobut-1-ene (CF_2_=CFCF_2_CF_2_H) with chlorine atoms in air. A mercury UV lamp was used to generate atomic chlorine, which initiated chemistry monitored by FTIR spectroscopy. Ethane was used as the reference compound for kinetic studies. Oxidation of heptafluorobut-1-ene initiated by a chlorine atom creates carbonyl difluoride (CF_2_=O) and 2,2,3,3 tetrafluoropropanoyl fluoride (O=CFCF_2_CF_2_H) as the major products. Anharmonic frequency calculations allowing for several low-energy conformations of 1,1,2,3,3,4,4 heptafluorobut-1-ene and 2,2,3,3 tetrafluoropropanoyl fluoride, based on density functional theory, are in good accord with measurements. The global warming potentials of these two molecules were calculated from the measured IR spectra and estimated atmospheric lifetimes and found to be small, less than 1.

## 1. Introduction

Saturated hydrofluorocarbons (HFCs) are non-ozone depleting substitutes for chlorofluorocarbons deprecated under the 1987 Montreal Protocol on Substances that Deplete the Ozone Layer, but they exhibit high global warming potentials (GWPs) and the Kigali Amendment adopted in 2016 outlines their phase down. Unsaturated HFCs offer more reactive alternatives, whose likely short atmospheric lifetimes would imply small GWPs. Because their GWPs are smaller than those for saturated HFCs by several orders of magnitude, and especially for fully fluorinated examples, several halogenated olefins are under consideration for practical applications [1]. This has motivated recent studies by several groups [2,3]. Here, we focus on an example of a fluorinated olefin, 1,1,2,3,3,4,4-heptafluorobut-1-ene (denoted as HFB) whose terminal pi bond offers a reactive site for radical attack. New data for its infrared (IR) spectrum, its likely atmospheric degradation products and its GWP are presented. We show how computed vibrational spectra can assist IR identification of novel species. We also measure the rate constant *k*_1_ for the reaction

HFB + Cl→products(1)

relative to *k*_2_ for

C_2_H_6_ + Cl→HCl + C_2_H_5_ (2)

and use this information to help assess the atmospheric lifetime of HFB.

## 2. Results

C_2_H_6_ was monitored by a band at 2868.0–2897.0 cm^−1^, and HFB by a band at 1767.0–1808.8 cm^−1^. Initial experiments with various concentrations prepared manometrically verified that the Beer–Lambert law was obeyed. We also verified that in the absence of UV light, no reaction occurred.

**Figure 1:**
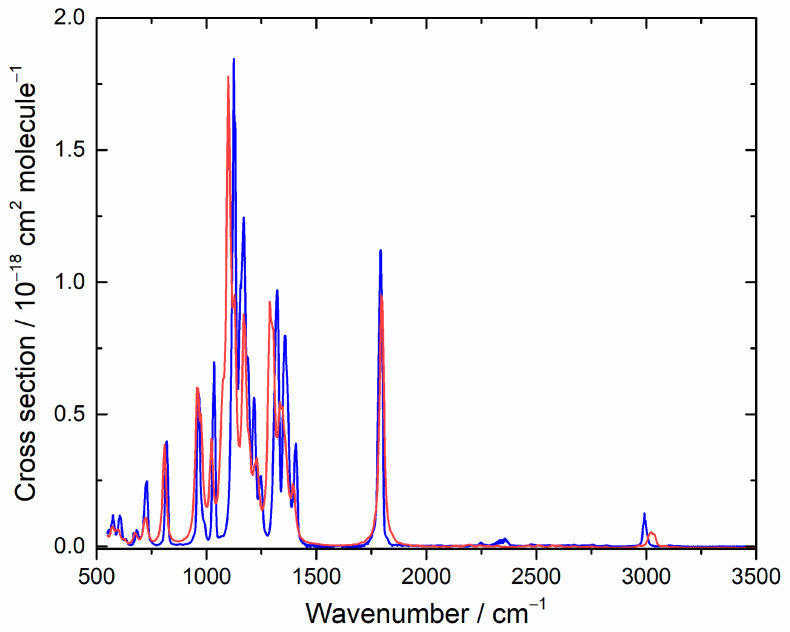
Measured (blue) and computed (red) IR spectra for heptafluorobut-1-ene. In the simulation, lines were broadened with a 16 cm^−1^ FWHM Lorentzian function, and the cross section is in arbitrary units.

**Figure 2:**
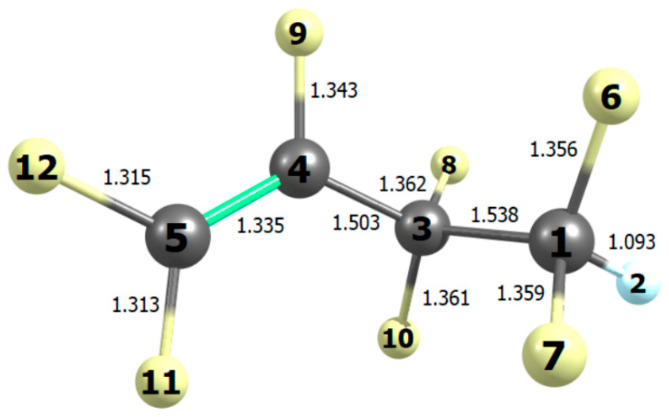
Bond lengths (10^−10^ m) in the most stable conformation of heptafluorobut-1-ene computed with B2PLYP/N07D theory.

The IR spectrum of HFB has not been reported before, and it is shown in Figure 1. Detailed cross sections are listed in Table S1 of the Supplementary Material. Figure 1 also shows the computed IR spectrum, which is in good accord. The simulated spectrum reflects the influence of 9 conformers, which are summarized in Table 1. There is 1 unique conformation and 4 degenerate pairs. Two of the degenerate pairs dominate at room temperature, conformers A and C. The corresponding dihedral angles refer to Figure 2 and describe torsions around the two C-C bonds.

Four kinetic runs were performed to measure *k*_1_. Different initial concentrations were employed, with equal pressures of HFB and ethane and an excess of chlorine. The conditions are summarized in Table 2. The temperature was 296 ± 2 K and the total pressure was 1 bar, made up with Ar. Successive spectra were captured approximately 40 s apart, each one with 25 co-added scans.

**Figure 3:**
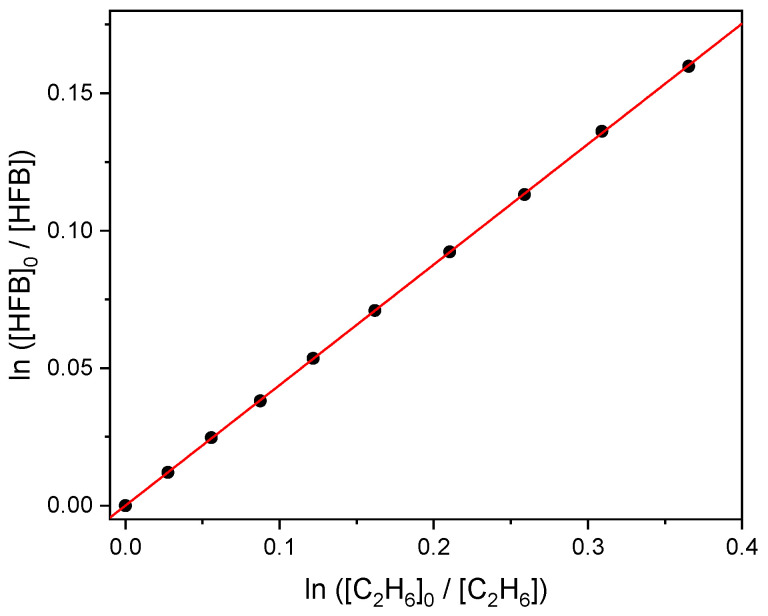
Relative consumption of heptafluorobut-1-ene and ethane by atomic chlorine.

Spectral subtraction from the initial conditions yields the ratios [HFB]_0_/[HFB] and [C_2_H_6_]_0_/[C_2_H_6_], and an example plot is shown in Figure 3. The slope of Figure 3 corresponds to the ratio *k*_1_/*k*_2_ (see Section 4.2). The intercepts of unconstrained linear fits were not significantly different from zero, so the intercepts were fixed at zero. We report 2σ statistical uncertainties in the slopes in Table 2. With *k*_2_ = (5.7 ± 0.6) × 10^−11^ cm^3^ molecule^−1^ s^−1^ [2], we estimate *k*_1_ = 2.5 × 10^−11^ cm^3^ molecule^−1^ s^−1^. We further allow for up to 3% systematic error in the pressure measurements, and with the 7% uncertainty in *k*_2_ propose a 95% confidence interval of ±0.2 × 10^−11^ cm^3^ molecule^−1^ s^−1^ for *k*_1_.

There are no prior determinations for comparison, but we note this value is similar to that for Cl + perfluorobut-1-ene. The latter compound, in which the terminal H atom of HFB is replaced by F, has a rate constant of (1.8 ± 0.4) × 10^−11^ cm^3^ molecule^−1^ s^−1^ [4]. If, instead, the C-H bond is retained and the C=C pi bond is replaced by a sigma bond, as in CF_3_CF_2_CF_2_CF_2_H (HFC-329p), then reactivity toward atomic Cl is 9 × 10^4^ times smaller than we find for HFB [5]. These comparisons suggest that the Cl chemistry of HFB starts at the pi bond.

Study of the products was carried out with two runs, where 10 back-to-back spectra with 185 co-added scans were obtained at intervals of 5 min. The initial reactant partial pressures are given in Table 3. The total pressure was made up to 1 bar with Ar.

**Figure 4:**
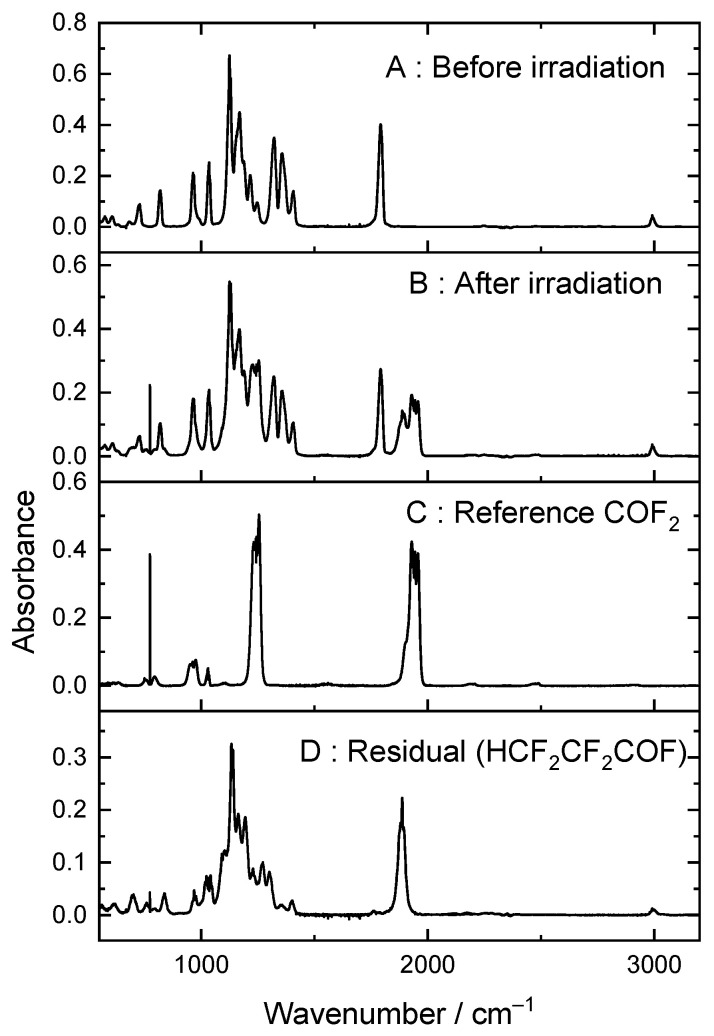
(**A**) Heptafluorobut-1-ene/Cl_2_/O_2_/Ar mixture before irradiation and (**B**) after irradiation, (**C**) a reference spectrum of COF_2_, and (**D**) the residual after subtracting unreacted HFB and COF_2_, assigned to tetrafluoropropanoyl fluoride (see text).

Figure 4 shows how during UV irradiation of these mixtures, new peaks appear. Some can be assigned to COF_2_, but there is a residual after accounting for COF_2_ and unreacted HFB.

**Figure 5:**
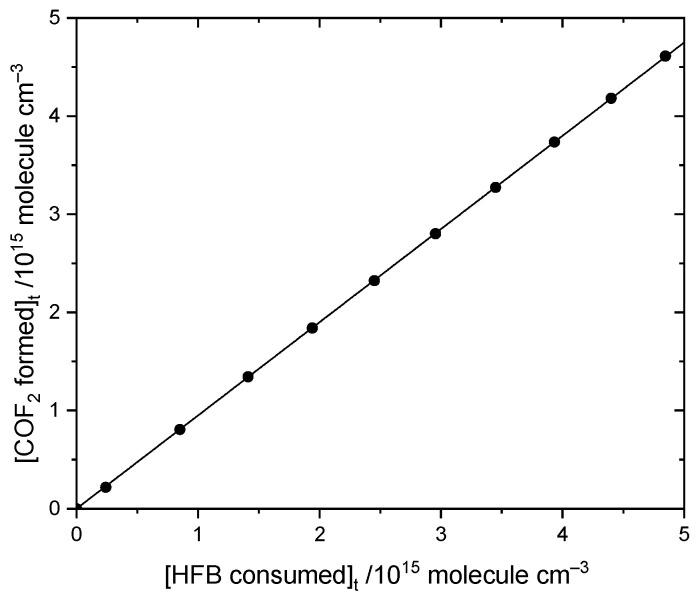
Comparison of COF_2_ formation to heptafluorobut-1-ene loss by reaction with atomic Cl in the presence of oxygen.

Focusing first on COF_2_, an example plot of its yield compared to consumption of HFB, Figure 5, has a slope of 0.95 ± 0.01. The second determination reproduced the same value. With allowance for uncertainties in our reference spectrum for COF_2_, we report 0.95 ± 0.07 for the yield of COF_2_. 

**Figure 6:**
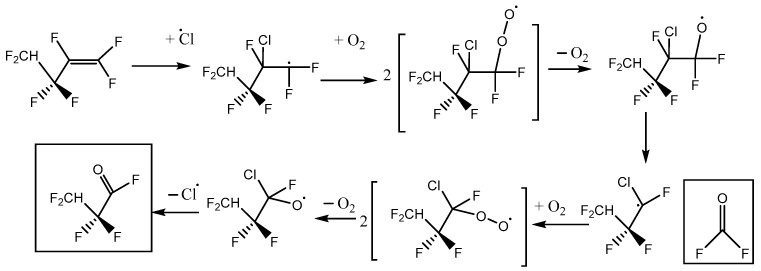
Reaction scheme A for heptafluorobut-1-ene reaction with atomic Cl in the presence of oxygen, with initial Cl attack at the inner C atom of the pi-bonded pair.

**Figure 7:**
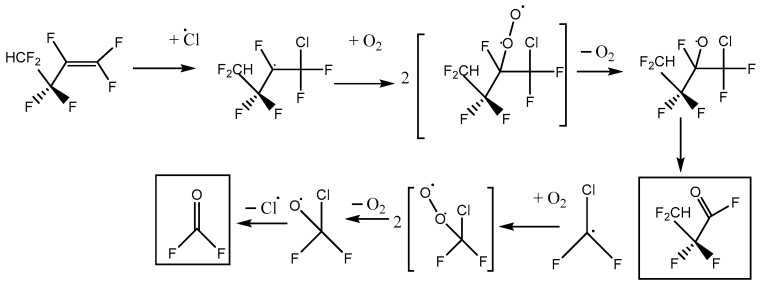
Reaction scheme B for heptafluorobut-1-ene reaction with atomic Cl in the presence of oxygen, with initial Cl attack at the outer C atom of the pi-bonded pair.

This is consistent with the following mechanisms in Figure 6 and Figure 7, which both imply a 1:1 COF_2_:HFB ratio. They follow a standard form, where oxygen adds to the initial radical created by Cl addition to make a peroxy species. In the atmosphere, peroxy radicals are typically converted to alkoxy radicals via O-atom abstraction by NO. In our laboratory experiments, there is no NO, but Cl atoms might play a similar role. The main route to alkoxy is probably through the self-reaction of pairs of peroxy molecules, which is feasible because their concentration is much higher than in the atmosphere. In these alkoxy radicals, a C-O pi bond can be made, accompanied by breaking the weakest sigma bond to the carbon atom. If a C-Cl bond is present, the Cl atom will be eliminated; otherwise, a C-C bond will break, i.e., there is elimination of a carbonyl compound (in these schemes, the acid fluorides COF_2_ or CF_2_HCF_2_COF). In both schemes, the same product 2,2,3,3-tetrafluoropropanoyl fluoride (or TPF) is predicted, so the question is: can the residual spectrum be rationalized as coming from TPF?

**Figure 8:**
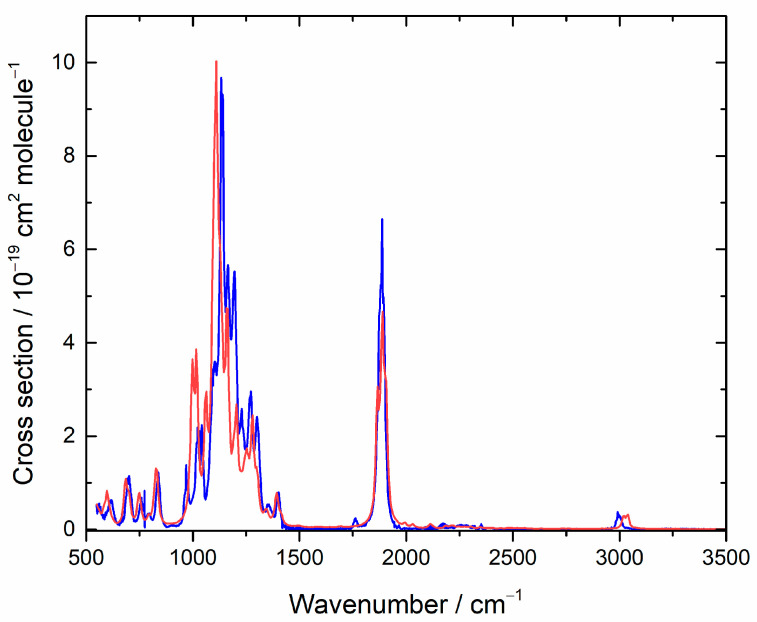
Measured (blue) and computed (red) IR spectra for tetrafluoropropanoyl fluoride. In the simulation, lines were broadened with a 16 cm^−1^ FWHM Lorentzian function, and the cross section is in arbitrary units.

To characterize the residual spectrum, we used the same procedure as described in Section 4.2, where data for various concentrations (based on an assumed unit yield) were combined at each frequency via the Beer–Lambert law to obtain the cross section. This spectrum is plotted in Figure 8, and σ values are provided in Table S2. An authentic spectrum of TPF is not available for comparison, so we apply our computational approach. Table 4 list the conformers we found and their relative Gibbs energies. As seen in Figure 8, agreement for the IR spectrum is good and consistent with TPF as the major co-product. Because typically the products of the Cl-initiated chemistry of organic compounds are similar to the products of OH-initiated processes, this product analysis gives insight into likely atmospheric degradation by OH, the dominant oxidizing agent.

**Table 4:** Computed properties of rotational conformers in tetrafluoropropanoyl fluoride.

Conformer	ΔG_298_^a^/kJ mol^−1^	Degeneracy	D1345 ^b^	D2134 ^b^	Weight
A	0	2	±108.7	∓172.9	0.46
B	3.32	1	0	180.0	0.06
C	1.66	2	±127.3	±67.3	0.24
D	1.65	2	±24.0	∓57.3	0.24

^a^ Computed Gibbs energy relative to the lowest conformer. ^b^ Dihedral angle in degrees (see Figure 9).

**Figure 9:**
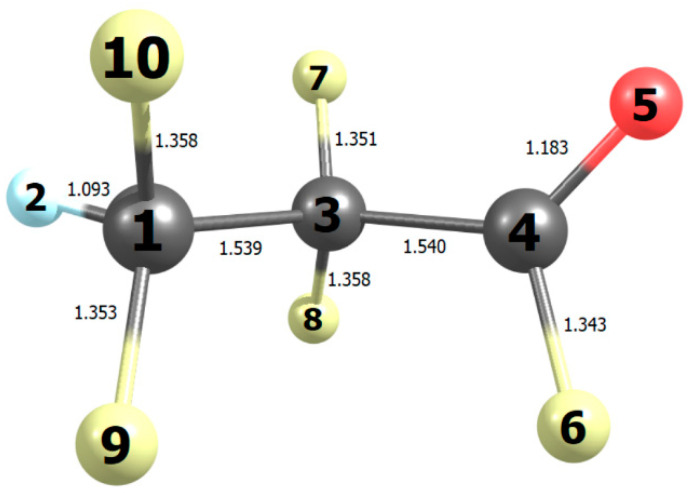
Bond lengths (10^−10^ m) in the most stable conformation of tetrafluoropropanoyl fluoride computed with B2PLYP/N07D theory.

**Figure 10:**
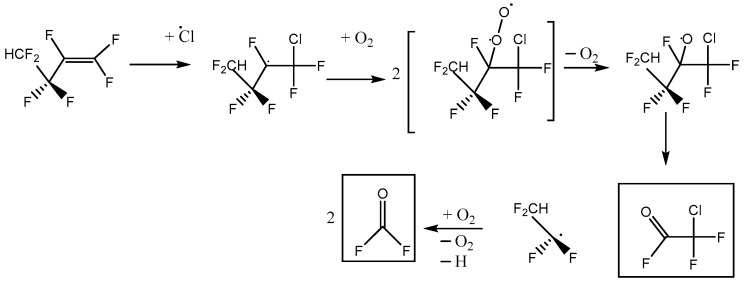
Alternative reaction scheme C for heptafluorobut-1-ene reaction with atomic Cl in the presence of oxygen, with initial Cl attack at the outer C atom of the pi-bonded pair.

We note that a third reaction scheme is, in principle, possible, illustrated in Figure 10. This pathway leads to the net formation of two molecules of COF_2_ per molecule of HFB, plus chlorodifluoroacetyl fluoride. No C-Cl stretches were observed, and the yield of COF_2_~1, so Scheme C plays a minor role at best. In the alkoxy radical that appears in Schemes A and C, CF_2_HCF_2_CFO·CF_2_Cl, either C-C bond next to the oxygen atom can break. Density functional theory indicates that dissociation to make CF_2_Cl (Scheme A) is ca. 6 kJ mol^−1^ more favorable than making CF_2_HCF_2_ (Scheme C). This intermediate thermochemistry might drive the branching ratio toward Scheme A.

## 3. Discussion

The average tropospheric concentration of Cl atoms, ca. 3 × 10^4^ molecule cm^−3^ [6], leads to a 1/e lifetime τ_Cl_ for destruction of HFB of 15 days. In the marine boundary layer, higher [Cl] may be expected, up to (1–3) × 10^5^ molecule cm^−3^ [7,8], which implies a shorter lifetime of 1.5–4.5 days and in this specialized environment can be the fastest removal pathway. The rate constant for HFB + OH is unknown, but it is likely similar to that for OH + perfluorobut-1-ene by analogy with the Cl-atom reactivity ratio discussed above, i.e., (1.9 ± 0.3) × 10^−12^ cm^3^ molecule^−1^ s^−1^ [4]. With an average tropospheric [OH] = 1 × 10^6^ molecule cm^−3^ [9], the lifetime for removal by OH alone is τ_OH_ = 6.1 days. The overall tropospheric lifetime of HFB, given by 1/τ = 1/τ_Cl_ + 1/τ_OH_, is τ = 4.3 days. TPF is an acid fluoride and is therefore likely to hydrolyze rapidly in liquid water. This process has been analyzed by Kotamarthi et al. for CF_3_COF [10]. They considered diffusion into clouds, then absorption into water droplets, followed by aqueous hydrolysis, and obtained a lifetime of 2.6 days. By analogy, we use τ = 2.6 days for TPF. A similar argument applies to COF_2_, so the ultimate products of HFB oxidation are soluble fluorinated carboxylic acids, which will be rained out.

**Figure 11:**
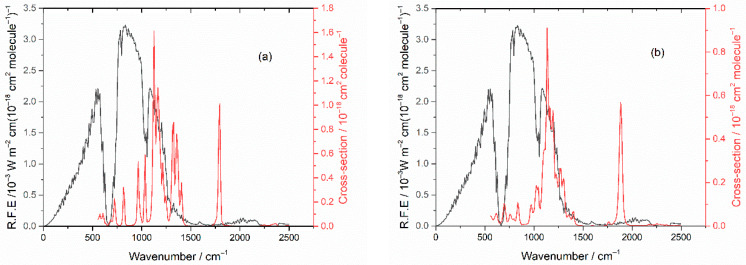
Earth’s radiation (RFE) plotted in black, left-hand scale. (**a**) Absorption cross sections of HFB in red, right-hand scale. (**b**) Absorption cross sections of TPF in red, right-hand scale.

There is significant overlap between the IR absorptions of HFB and TPF and the blackbody radiation emitted from the Earth (the radiative forcing efficiency, RFE), especially in the region near 1200 cm^−1^ arising from C-F stretching modes, as seen in Figure 11. We use the method of Hodnebrog et al. [11] to derive the radiative efficiency (RE) and obtain RE = 0.29 and 0.18 W m^−2^ ppb^−1^ for HFB and TPF, respectively. For comparison, our RE value for HFB is similar to the value of 0.30 recommended for perfluorobut-1-ene [11]. Inclusion of the correction *f*(τ) for non-homogeneous vertical profiles of short-lived species [11] yields final values of RE = 0.013 and 0.0052 W m^−2^ ppb^−1^ for HFB and TPF, respectively.

The GWP reflects the integrated IR absorbance and induced temperature change relative to CO_2_ on a per mass basis for a given time period. Over a 20-year time horizon, the GWP_20_ values for HFB and its decomposition product TPF are both small, at 0.36 and 0.07, respectively. Of course, extended horizons yield smaller values, and the GWP_100_, to the extent this is meaningful for short-lived species, is approximately 0.01–0.02 for both species.

## 4. Materials and Methods

### 4.1. Materials

**Figure 12:**
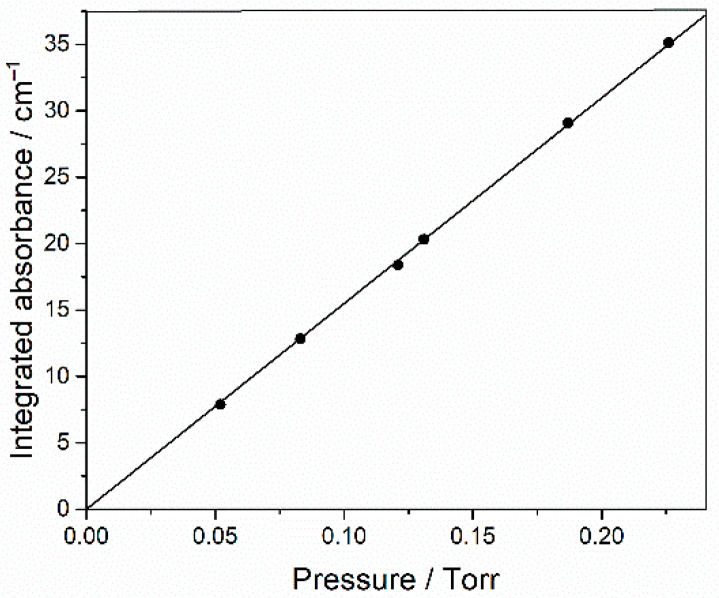
Integrated base 10 absorbance of 1968.75–1907.75 cm^−1^ band of COF_2_ as a function of its partial pressure (1 Torr = 1.333 mbar), made up to 1 bar with Ar.

The reagents used were HFB (Synquest, Alachua, FL, USA, 97% purity), ethane (Praxair, Danbury, CT, USA, 99.9%) and Cl_2_ (Aldrich, St. Louis, MO, USA, >99.5% purity), which we purified by freeze-pump-thaw cycles with liquid nitrogen. O_2_ and Ar (Air Liquide, Pasadena, TX, USA, >99.99% purity) were used directly from their cylinders. Carbonyl difluoride was photochemically synthesized from a mixture of 13 mbar perfluorobutadiene, 7 mbar Cl_2_ and 130 mbar O_2_, and then unreacted starting materials and side products were trapped with a pentane/liquid nitrogen slush at 143 K. The integrated absorbance of a strong band at 1968.75–1907.75 cm^−1^ was measured for several partial pressures of COF_2_, made up to 1 bar with Ar. The proportionality shown in Figure 12 confirms the applicability of the Beer–Lambert law and the slope yields an integrated band strength (base e) of (4.56 ± 0.05) × 10^−17^ cm molecule^−1^.

### 4.2. Experimental Method

We use IR spectroscopy to probe the chemistry initiated by photolytically generated chlorine atoms, evaluate reaction kinetics, identify products and quantify product yields. Our apparatus has been described in detail elsewhere [12]. In overview, mixtures of HFB and molecular chlorine diluted in a large excess of argon bath gas at 1 bar pressure and room temperature were exposed to steady UV illumination at 365 nm from a mercury lamp over time scales of 10–50 min. Experiments were carried out in a long-path multipass cell (ℓ = 240 cm) mounted inside the sample compartment of a Nicolet iS50 FT-IR spectrometer (Thermo Fisher Scientific, Madison, WI, USA). Sequential spectra were recorded at 1 cm^−1^ resolution with a mercury cadmium telluride detector (Thermo Fisher Scientific, Madison, WI, USA) cooled with liquid nitrogen, as a function of time while HFB was depleted by reaction 1.

For relative-rate kinetics measurements, ethane as a reference compound was added to the initial mixture, and it was also depleted with time, via reaction 2. With *k*_1_ and *k*_2_ as the bimolecular rate constants for reactions 1 and 2, integration of the ratio of the rate laws yields the standard result [13].

(3) $$\ln\left(\frac{{\left[\mathrm{HFB}\right]}_{0}}{\left[\mathrm{HFB}\right]}\right)=\frac{k_{1}}{k_{2}}\;\ln\left(\frac{{\left[C_{2}H_{6}\right]}_{0}}{\left[C_{2}H_{6}\right]}\right)$$

Because *k*_2_ is known, *k*_1_ is obtained from measurements of the concentrations of HFB and C_2_H_6_ ratioed to their initial values, as evaluated by spectral subtraction.

For product studies, no ethane reference is used, molecular oxygen is added to simulate atmospheric conditions, and product concentrations are obtained as a function of the amount of HFB consumed via the IR spectrum. When the base 10 absorbance *A* and concentration *c* are known, in terms of the Beer–Lambert law, we may write

σ = 2.3026 *A*/(*c* ℓ) (4)

where, with the path length ℓ in cm and *c* in molecule cm^−3^, the base e cross section σ is in cm^2^ molecule^−1^. We determine σ at every discrete frequency in the absorption spectrum from the slope of a linear plot of measured *A* vs. several values of *c*.

### 4.3. Computational Method

Geometries and frequencies of target molecules were obtained via density functional theory with the B2-PLYP functional [14,15] combined with the N07D basis set developed by Barone and coworkers for vibrational analysis [16,17]. Calculations were made with the Gaussian16 program suite [18]. Rotation around sigma bonds leads to different local minima, which were systematically optimized. Predictions of the IR spectrum are made via second-order vibrational perturbation theory [17], which accounts for anharmonicity in the fundamental vibrational modes without empirical scale factors, and, further, incorporates contributions of overtones and combination bands. IR intensity data for each conformer were weighted by the relative abundance at *T* = 298 K. This was derived from the degeneracy of the given conformer energy, *n*, and the computed Gibbs energy, Δ*G_i_*, relative to the most stable conformer, as the ratio of *n*_i_ exp(−Δ*G*_i_/R*T*) to the sum of all conformer terms.

## 5. Conclusions

For HFB, the IR absorption cross sections and reactivity toward Cl atoms have been characterized, as well as the main products in the presence of oxygen, carbonyl difluoride and TPF. Anharmonic IR spectra computed over Boltzmann distributions of conformers for HFB and TPF agree with observations and help assign TPF as a major product. The measurements yield small values for the GWP_20_, below 1. Because the atmospheric lifetimes are a few days, detailed evaluation is a function of local conditions, but the results suggest that emissions of HFB would have a negligible impact on climate change.

## Figures and Tables

**Table 1:** Properties of rotational conformers in heptafluorobut-1-ene.

Conformer	Δ*G*_298_^a^/kJ mol^−1^	Degeneracy	D1345 ^b^	D2134 ^b^	Weight
A	0	2	±97.3	±178.5	0.58
B	6.58	1	0	180.0	0.02
C	1.26	2	±124.9	±61.6	0.35
D	9.18	2	±14.0	∓62.2	0.01
E	6.70	2	±98.9	∓66.3	0.04

^a^ Computed Gibbs energy relative to the lowest conformer. ^b^ Dihedral angle in degrees (see Figure 2).

**Table 2:** Condition for relative rate measurements of Cl atoms with heptafluorobut-1-ene (*k*_1_) vs. C_2_H_6_ (*k*_2_).

*p* Cl_2_/mbar	*p* HFB/mbar	*p* C_2_H_6_/mbar	*k*_1_/*k*_2_
0.81	0.27	0.27	0.44 ± 0.01
2.14	0.47	0.47	0.44 ± 0.01
1.80	0.54	0.54	0.44 ± 0.01
1.80	0.49	0.49	0.46 ± 0.01

**Table 3:** Initial conditions for product studies in the Cl/heptafluorobut-1-ene/O_2_ system.

*p* Cl_2_/mbar	*p* HFB/mbar	*p* O_2_/mbar
0.13	0.22	25
0.21	0.32	32

## Data Availability

The data presented in this study are available on request from the corresponding author.

## Supplementary Materials

The following supporting information can be downloaded online, Table S1: IR absorption cross sections for absorption by 1,1,2,3,3,4,4-heptafluorobut-1-ene; Table S2: IR absorption cross sections for absorption assigned to 2,2,3,3-tetrafluoropropanoyl fluoride.

## Sample Availability

Not available.
